# On the Functions and Values of Journalism

**DOI:** 10.1007/s13347-026-01127-z

**Published:** 2026-06-12

**Authors:** Rubén Marciel

**Affiliations:** Department of Political Science and International Relations, University of Geneva, Bd. du Pont-d’Arve 40, 1205 Genève. Office 5363, Geneva, Switzerland

**Keywords:** Journalism, Deliberation, Legitimacy, Stability, News media, Media policy

## Abstract

In his comment to my article ‘Private Media, Public Funds, and Democratic Allocation’, Fox raised two pressing challenges. The first challenge is theoretical and claims that the deliberative view of journalism I endorse, which assumes as its only function the promotion of deliberation, is too narrow to incorporate all that makes journalism valuable. The second challenge, which naturally follows from the first one, is institutional: such a narrow view of journalism leads to inadequate eligibility criteria, as these criteria will trace back only the institutional features that predispose media organizations to promote deliberation. In this reply, I defend my view of journalism against these two challenges.

Carl Fox is one of the most authoritative voices in the blossoming politico-philosophical debate on news media and journalism, to which he has contributed like few others.^1^ In his comment on my article on civic media policies (Marciel (2026a); Fox (2026) raises two pressing and interrelated challenges which I wish to address in this short reply.

The first challenge is theoretical and claims that the deliberative view of journalism I endorse there is too narrow to capture all what makes journalism valuable. In my view (see Marciel 2025, 2026b), journalism’s main function is to promote high-quality democratic deliberation. This approach, Fox notes, is “monistic” (2026. p. 7) as it assumes that journalism serves only one function—that of informing the public—and that its value ultimately derives from a single political value: legitimacy. And this is problematic because the “narrowness of the deliberative ideal” (p. 7) leads to neglecting other important values and functions. In particular, Fox calls our attention to political stability, understood as a property of democratic polities that, as he writes elsewhere, “depends on the degree to which its citizens are disposed to play fair with one another and to refrain from imposing their particular conceptions of the good on everyone” (Fox, 2023, p. 146; cf. Fox, 2026, p. 6). In his view, journalism should promote stability too, and not just legitimacy.

Against this challenge, I wish to suggest two ways in which a monistic deliberative account of journalism like mine could actually accommodate the value of stability.

The first way requires distinguishing between the functions of journalism and the values those functions serve. Journalistic functions are the tasks journalists are expected to perform in discharging their role-based obligations. By contrast, the values journalism serves are the important goods that these functions protect or help realize. Crucially, a single function may serve several values. For instance, the watchdog function might be said to serve the values of democratic accountability, efficiency, equality, and even stability as defined by Fox. After all, by helping hold public officials accountable, journalism may reinforce citizens’ confidence that democratic norms are respected and that violations are sanctioned, thereby strengthening their willingness to cooperate within democratic institutions. Likewise, promoting good deliberation might be said to serve not only legitimacy, but also a range of democratic values that include stability. Deliberation requires citizens to relate to one another as free and equal persons, recognizing others as legitimate participants in public reasoning and as bearers of equal rights. Thus, if journalism successfully promotes deliberation, it may also strengthen citizens’ commitment to democratic norms and contribute to political stability.

A second way to incorporate stability within a monistic account of deliberative journalism requires distinguishing between first-order and second-order functions. First-order functions are those activities for whose realization the institution exists. Second-order functions, by contrast, are subsidiary activities required for the institution to effectively fulfill its first-order function(s). Within the deliberative ideal I endorse, promoting deliberation is indeed journalism’s single first-order function. This is, however, compatible with recognizing several second-order functions. For instance, deliberative journalists may need to generate revenue to secure the resources necessary to promote deliberation effectively; in that sense, revenue making could be understood as a second-order function of deliberative journalism (Marciel 2026b, sec. 4.4). Something similar may be said about the promotion of stability. To the extent that citizens’ willingness to play fair, abide by democratic rules, and endorse basic democratic values is necessary for quality deliberation to occur, the promotion of stability might be considered a second-order function of deliberative journalism, one that should be fulfilled subsidiarily to ensure the fulfillment of the first-order function.

Someone concerned about democratic stability, as Fox is, might worry that this unjustifiably renders stability a second-class value. Note, however, that the issue here is not the comparative worth of democratic values in general, but rather their relative importance for journalism as an institution. Claiming that journalism should be more concerned with promoting deliberation leading to legitimate decisions than with promoting democratic stability does not imply that democratic legitimacy is intrinsically more valuable than democratic stability. It merely means that journalists, given their institutional role, should prioritize one over the other.

The reason for placing stability below legitimacy in the ranking of values that matter for journalism is that other institutions—such as schools, political parties, and representative institutions—are at least as well positioned, if not better positioned, to cultivate citizens’ willingness to play fair and abide by democratic rules. By contrast, it is less realistic to expect these institutions to promote high-quality deliberation in the way journalists can and should do. It therefore seems more efficient to let journalists concentrate on promoting deliberation—the task for which they are better equipped than any other institution—while treating as second-order functions those tasks that other institutions are better suited to promote.

This leads to the second challenge, which concerns the eligibility criteria that media organizations must satisfy in order to qualify for public funding. As Fox argues, an excessively narrow conception of journalism—one that focuses only on part of what journalists should do and part of what makes journalism worthwhile—would generate inadequate eligibility criteria, since such criteria would reflect only part of what good journalism is about.

However, if what I have argued so far is correct, the problem is a bit less serious than it may initially appear. What we need is a full-fledged account of what “deliberative journalism” entails, one that illuminates the intricate connections between, on the one hand, journalism’s first-order function and its second-order functions and, on the other, all these first- and second-order functions and the different values they are meant to serve. Once these connections are explicated, it should be possible to develop accurate and well-tailored eligibility criteria incorporating all relevant concerns, including those related to stability. Of course, this is no easy task. But it seems possible. For instance, concerns about stability could be partially incorporated into civic media policies by rendering ineligible those outlets that spread forms of speech that undermine democracy—such as fake news, hate speech, or, perhaps more aptly, what Fox (2023) calls “bad disruptive speech.”

## Data Availability

Not applicable.

## Footnotes

1 He is the author of a pioneering article that develops a Rawlsian account of journalistic objectivity (Fox, 2013) and the co-editor of (and contributor to) the two most important books on political philosophy and news media so far (Fox & Saunders 2019, 2024).
